# STIM/Orai-Mediated Store-Operated Ca^2+^ Entry in the Pathogenesis of Fibrosis: Mechanisms and Therapeutic Opportunities

**DOI:** 10.3390/cells15110980

**Published:** 2026-05-26

**Authors:** Yang Yi, Md Nasim Uddin, Keira Killeen, Donald L. Gill, Yandong Zhou

**Affiliations:** Department of Cell and Biological Systems, Penn State College of Medicine, Hershey, PA 17033, USA; yqy5526@psu.edu (Y.Y.); mfu5071@psu.edu (M.N.U.); krk5787@psu.edu (K.K.); dongill@psu.edu (D.L.G.)

**Keywords:** STIM, Orai, SOCE, fibrosis, calcium

## Abstract

Store-operated calcium entry (SOCE), mediated by the endoplasmic reticulum (ER) Ca^2+^ sensors stromal interaction molecule (STIM) proteins and the plasma membrane (PM) Orai channels, is essential for calcium signaling and a wide range of physiological processes. Precise regulation of SOCE is critical for maintaining tissue homeostasis, whereas its dysregulation contributes to diverse pathological conditions, particularly organ fibrosis. In this review, we outline the molecular basis of SOCE and discuss how its dysregulation is implicated in human disease. We further emphasize the pivotal role of SOCE in driving fibrotic progression across major organ systems. Finally, we summarize current therapeutic strategies targeting SOCE and highlight their potential for the treatment of fibrosis.

## 1. Introduction

Calcium is a universal intracellular messenger that coordinates gene expression, proliferation, migration, and contraction [1,2]. Accordingly, the precise spatiotemporal regulation of Ca^2+^ signaling is crucial for maintaining cellular and tissue homeostasis [3,4]. Store-operated calcium entry (SOCE) is a central mechanism responsible for generating sustained elevations in cytosolic Ca^2+^ [5,6]. It is triggered by depletion of endoplasmic reticulum (ER) Ca^2+^ stores and is molecularly orchestrated by the ER-resident Ca^2+^ sensor stromal interaction molecule (STIM) and the plasma membrane Ca^2+^ channel Orai [7,8]. Upon ER Ca^2+^ store depletion, STIM proteins oligomerize and translocate to ER–plasma membrane junctions, where they directly gate Orai channels to mediate Ca^2+^ influx for store refilling and sustained downstream signaling [9,10]. Through this process, the STIM/Orai axis replenishes ER Ca^2+^ stores and generates prolonged Ca^2+^ signals that regulate key functions in diverse cell types, including immune cells, muscle cells, neurons, and secretory cells [11].

Tight regulation of SOCE is essential for physiological homeostasis [3,4]. Increasing evidence indicates that SOCE dysregulation can arise from altered STIM/Orai expression, pathogenic mutations, or aberrant channel activation. Consequently, dysregulated SOCE has been implicated in the pathogenesis of multiple disorders, including cancer [12,13,14], immunodeficiencies [15,16], neurodegenerative diseases [17,18,19], and vascular pathologies [20,21]. Notably, SOCE dysregulation is increasingly recognized as a major contributor to organ fibrosis [22,23,24,25]. Fibrosis, characterized by excessive accumulation of extracellular matrix components, is a common pathological consequence of chronic tissue injury and inflammation in vital organs, including the heart [23], lungs [24], liver [25], and kidneys [26]. This process leads to progressive tissue scarring, structural distortion, and ultimately organ failure [27], thereby contributing substantially to global morbidity and mortality [28,29].

Current anti-fibrotic therapies, including the FDA-approved drugs pirfenidone and nintedanib, are often limited in efficacy and may be associated with significant systemic side effects [30,31]. These limitations make them suboptimal for long-term disease management. Therefore, new therapeutic strategies that target the core pathological mechanisms of fibrosis are urgently needed [32,33]. In fibrotic diseases, persistent activation of key effector cells, including myofibroblasts and pro-fibrotic immune cells, is driven by a complex signaling network [34,35]. SOCE represents a central regulatory mechanism within this network and promotes essential fibrogenic processes, including fibroblast-to-myofibroblast differentiation, proliferation, migration, and excessive extracellular matrix production [36]. Given the central role of SOCE in fibrosis and the well-defined nature of its core molecular components, the development of small-molecule inhibitors targeting the SOCE pathway represents a promising and potentially precise therapeutic strategy [37]. Such inhibitors may enable intervention at critical points in fibrotic progression while reducing the off-target adverse effects often associated with conventional nonspecific therapies [38].

This review aims to systematically delineate the molecular architecture and regulatory mechanisms of the SOCE pathway. We then examine the pivotal contribution of SOCE dysregulation to fibrotic progression across major organ systems. Finally, we evaluate emerging therapeutic strategies targeting SOCE components and their regulatory networks, with particular emphasis on the progress and challenges associated with the development of novel small-molecule inhibitors. Through this synthesis, we aim to provide a rational framework and future directions for the development of SOCE-targeted anti-fibrotic interventions.

## 2. Molecular Basis of SOCE

The molecular identity of SOCE remained a major unresolved question in signal transduction until the landmark discovery of two core protein families: STIM, the endoplasmic reticulum (ER)-luminal Ca^2+^ sensor, and Orai, the pore-forming subunit of calcium release-activated calcium (CRAC) channels, which are the principal channels mediating SOCE in the plasma membrane (PM).

### 2.1. STIM1

Human STIM1 is a 685-amino acid type I transmembrane protein, originally identified as a 90 kDa phosphoprotein expressed in a variety of human primary cells [39,40]. Structural studies have revealed multiple functional domains in its N-terminal region, including an ER signal peptide (aa 1–22), a canonical EF-hand Ca^2+^-binding motif (aa 63–96), an adjacent hidden EF-hand (aa 97–128), and a sterile α-motif (SAM; aa 132–200), which plays an essential role in protein–protein interactions [41,42]. The transmembrane domain (aa 214–234) is highly conserved across the STIM protein family. Downstream of this region, the cytosolic portion contains three coiled-coil domains—CC1 (aa 238–342), CC2 (aa 364–389), and CC3 (aa 399–423)—as well as a CRAC modulatory domain (aa 470–491), a proline/serine-rich region (aa 600–629), and a polybasic lysine-rich region (aa 671–685) [43,44]. The STIM1-Orai activating region (SOAR; aa 344–442) has been identified as the key STIM1 domain responsible for coupling to and activating Orai channels [43]. Crystallographic studies further revealed that the SOAR domain adopts a dimeric conformation [45,46].

### 2.2. Orai1

In many cell types, depletion of ER Ca^2+^ stores activates Ca^2+^-selective channels in the plasma membrane [47]. These channels, whose Ca^2+^-conducting pore is formed by Orai proteins, mediate store-operated Ca^2+^ entry. Humans possess three Orai isoforms: Orai1–3. Among them, Orai1 is a 301-amino acid protein with four transmembrane domains (TM1–TM4), with both its N- and C-termini located in the cytosol [48]. The crystal structure of Drosophila melanogaster Orai has provided important insights into ion permeation, selectivity, and gating in CRAC channels. This structure, which shares 73% sequence identity with human Orai1 within the transmembrane region [49], supports a hexameric channel stoichiometry, with six subunits assembled around a central pore.

### 2.3. STIM1/Orai1 Activation of SOCE

The canonical SOCE pathway is triggered by agonist-induced depletion of ER Ca^2+^ stores, which promotes Ca^2+^ dissociation from STIM proteins and initiates their oligomerization and translocation to specialized ER–PM junctions. At these sites, direct STIM–Orai coupling activates Orai channels, leading to tightly regulated and sustained extracellular Ca^2+^ influx [50].

The STIM1–Orai1 interaction has been characterized by NMR. The binding interface, termed the STIM1–Orai1 association pocket (SOAP), involves key residues from both proteins, including positively charged residues (K382, K384, K385, and K386) and hydrophobic/aromatic residues (L347, Y361, Y362, L373, A376, and L351) in STIM1, as well as residues L273, L276, R281, L286, and R289 in the Orai1 C-terminus [51]. Beyond this direct binding interface, subsequent studies revealed that STIM1-mediated activation of Orai1 depends on a remote allosteric gating mechanism. In this model, STIM1 binding to the Orai1 C-terminal/TM4 nexus induces conformational rearrangements that are transmitted across the channel to open the central TM1 pore, thereby coupling intermembrane binding to channel gating [52].

More recent work has further refined this mechanism by identifying a critical Phe–His pair within the apical region of STIM1 SOAR [53]. In particular, the outer F394–H398 pair plays a dual role in STIM1 activation and Orai1 gating. In resting STIM1, this locus is buried within the folded CC1-clamped conformation; upon store depletion, it becomes exposed, allowing F394 to contribute to Orai1 coupling, whereas H398 is required for efficient transduction of STIM1 binding into Orai1 channel opening. These findings indicate that the SOAR apex is not only part of the binding surface for Orai1 but also a key determinant of the conformational coupling mechanism that links STIM1 activation to Orai1 channel gating.

### 2.4. Other STIM and Orai Isoforms

In addition to STIM1 and Orai1, mammalian cells express several related isoforms that further diversify SOCE. Among STIM proteins, STIM2 is closely related to STIM1 but generally responds to smaller reductions in ER Ca^2+^, suggesting a stronger role in maintaining basal Ca^2+^ homeostasis and fine-tuning SOCE [54,55,56]. Alternative splicing of STIM2 generates at least two major variants, STIM2.1 (STIM2β) and STIM2.2 (STIM2α), which differ functionally: STIM2.2 generally supports SOCE, whereas STIM2.1 is considered a weaker activator or even a negative modulator of Orai channel activation [57,58]. STIM1 also exists in multiple forms, including STIM1L [59]. Compared with the short canonical form, STIM1L contains an additional sequence that enables faster coupling to Orai channels and more rapid SOCE activation in some cell types.

Orai family members also include Orai2 and Orai3, both of which can participate in store-operated Ca^2+^ entry, although their channel properties differ from those of Orai1. In particular, Orai3 displays distinct gating and pharmacological behavior and can also contribute to store-independent Ca^2+^ entry pathways [60,61]. Overall, unlike the well-defined STIM1/Orai1 pair, the structural basis and regulatory mechanisms of these alternative STIM and Orai isoforms remain incompletely understood. Further studies are needed to define how STIM2-, Orai2-, and Orai3-containing complexes contribute to Ca^2+^ signaling under physiological conditions and in pathological processes such as fibrosis.

## 3. Regulation of STIM1/Orai1-Mediated SOCE

**Figure 1:**
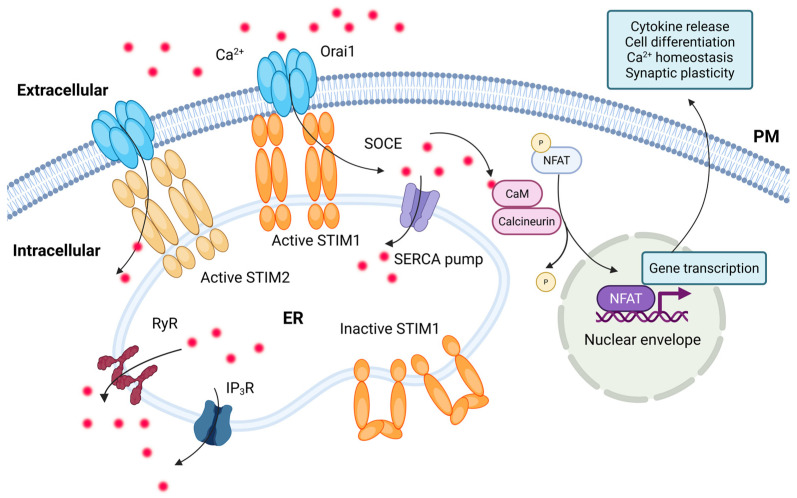
Key steps of SOCE. Agonist stimulation triggers Ca^2+^ release from the endoplasmic reticulum (ER) through IP_3_Rs and/or RyRs, leading to ER Ca^2+^ store depletion. STIM1 then translocates to ER–plasma membrane (PM) junctions, where it activates Orai1 channels to initiate store-operated Ca^2+^ entry (SOCE). The elevated cytoplasmic Ca^2+^ is subsequently pumped back into the ER by the SERCA pump, helping to replenish ER Ca^2+^ stores and sustain Ca^2+^ signaling through IP_3_Rs/RyRs. Meanwhile, Ca^2+^ entering through Orai1 binds to CaM, and Ca^2+^-bound CaM activates CN. Activated CN dephosphorylates NFAT, promoting its nuclear translocation and transcriptional activity. Abbreviations: ER, endoplasmic reticulum; IP_3_R, inositol 1,4,5-trisphosphate receptor; PM, plasma membrane; RyR, ryanodine receptor; SERCA, sarcoplasmic/endoplasmic reticulum Ca^2+^-ATPase; SOCE, store-operated Ca^2+^ entry; STIM1, stromal interaction molecule 1; CaM, calmodulin; CN, calcineurin; NFAT, nuclear factor of activated T cells. Figure created with BioRender.com.

Store-operated calcium entry is not a uniform signaling pathway; rather, its magnitude, kinetics, and downstream consequences are finely tuned according to cell type and physiological context. STIM1/Orai1-mediated SOCE is regulated by a multilayered network that includes intracellular Ca^2+^ store dynamics, downstream Ca^2+^-responsive signaling pathways, accessory proteins, and post-translational modifications [62] (Figure 1). These regulatory mechanisms not only determine the extent of Ca^2+^ influx but also shape the specificity of SOCE-dependent biological responses.

### 3.1. NFAT

Nuclear factor of activated T cells (NFAT) was originally identified as an inducible transcription factor that binds to the interleukin-2 (IL-2) promoter in activated T cells [63]. SOCE is a major upstream activator of NFAT signaling. Sustained Ca^2+^ influx through Orai channels promotes calcineurin-dependent dephosphorylation of NFAT, which triggers its nuclear translocation and subsequent activation of NFAT-dependent gene expression [64]. This signaling axis is essential for a wide range of cellular processes, including cytokine production, cell differentiation, Ca^2+^ homeostasis, and synaptic plasticity [65]. Consistent with this central role, loss-of-function mutations in Orai1 or STIM1 impair NFAT activation and contribute to human disease, particularly immunodeficiency [66].

### 3.2. RyR, IP_3_R and SERCA Pump

The activity of STIM1/Orai1-mediated SOCE is tightly coupled to the dynamic regulation of intracellular Ca^2+^ stores. Ryanodine receptors (RyRs) and inositol 1,4,5-trisphosphate receptors (IP_3_Rs) mediate Ca^2+^ release from the endoplasmic or sarcoplasmic reticulum, thereby contributing to store depletion and subsequent activation of STIM1 [67,68,69]. In contrast, the sarcoplasmic/endoplasmic reticulum Ca^2+^-ATPase (SERCA) restores luminal Ca^2+^ levels by pumping cytosolic Ca^2+^ back into the ER/SR [70]. Through this balance between Ca^2+^ release and reuptake, RyRs, IP_3_Rs, and SERCA influence the threshold, amplitude, and duration of SOCE. Thus, STIM1/Orai1 signaling is functionally integrated with intracellular Ca^2+^ store handling rather than acting as an isolated influx pathway.

### 3.3. Accessory Proteins and Post-Translational Regulation

In addition to intracellular Ca^2+^ store dynamics, STIM1/Orai1-mediated SOCE is further tuned by accessory proteins [71,72,73,74,75,76] and post-translational modifications [77]. These mechanisms can influence STIM1 conformational rearrangement, puncta formation, STIM1–Orai1 coupling, and signal termination. Recent preprint evidence identified the glucosidase II complex, consisting of GANAB and PRKCSH, as a novel regulator of STIM1 activation dynamics, suggesting that early stages of STIM1 activation are subject to additional layers of molecular control [78]. Phosphorylation [79] and ubiquitination [80] of SOCE components may further regulate their localization, stability, and functional activity in a context-dependent manner. Because some of these findings are still emerging, additional studies will be needed to clarify their precise mechanisms and physiological relevance.

## 4. SOCE in Human Disease: Mechanisms Relevant to Fibrosis

**Figure 2:**
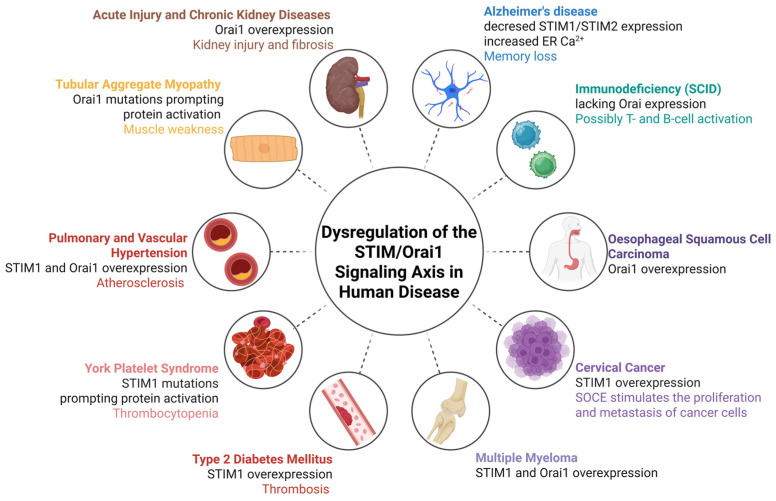
Disease relevance of STIM/Orai-mediated Ca^2+^ signaling. Dysregulation of STIM/Orai-dependent store-operated Ca^2+^ entry (SOCE) contributes to diverse human diseases, including kidney injury and fibrosis [90], tubular aggregate myopathy [91], pulmonary/vascular hypertension and atherosclerosis [92,93], York platelet syndrome [94,95], type 2 diabetes-associated thrombosis [96,97], immunodeficiency/SCID [98,99], Alzheimer’s disease [17,100], and several cancers [101], including oesophageal squamous cell carcinoma [102], cervical cancer [103], and multiple myeloma [104]. Depending on the disease context, either gain or loss of STIM/Orai function alters Ca^2+^-dependent signaling pathways that regulate fibrosis, muscle function, platelet activation, immune responses, neuronal function, and cancer progression. Figure created with BioRender.com.

As a major mechanism underlying sustained cytosolic Ca^2+^ signaling, SOCE plays an important and multifaceted role in the pathogenesis of a wide range of human diseases. By mediating prolonged Ca^2+^ influx following depletion of endoplasmic reticulum (ER) Ca^2+^ stores, SOCE regulates diverse cellular processes, including gene expression, proliferation, differentiation, and migration. Dysregulation of this pathway has been increasingly implicated in disease initiation and progression, particularly in cancer and fibrosis. In this review, we focus on disease settings most relevant to fibrotic remodeling (Figure 2), including cardiovascular disorders such as cardiac fibrosis and vascular stiffening, cancer-associated stromal remodeling characterized by activated fibroblasts and desmoplastic reactions, and chronic inflammatory conditions involving pulmonary, renal, and hepatic fibrosis [25,81,82,83,84,85,86,87,88,89]. These pathological contexts share common features, including persistent fibroblast activation and excessive extracellular matrix deposition, and together provide important mechanistic insight into how aberrant STIM/Orai signaling may drive a pro-fibrotic phenotype. Specifically, dysregulated SOCE can promote fibroblast-to-myofibroblast transition, enhance the production and crosslinking of extracellular matrix proteins such as collagen and fibronectin, and ultimately contribute to progressive tissue scarring and organ dysfunction.

### 4.1. Cardiovascular Remodeling and Fibrosis-Related Disease

Dysregulation of the SOCE pathway disrupts intracellular Ca^2+^ homeostasis and contributes to vascular and cardiac pathology. This pathogenic axis has been implicated in a variety of clinical and experimental settings, including hypertension, rare genetic disorders, metabolic disease, and fibrotic cardiovascular conditions. In hypertension, increased STIM1 and Orai1 expression contribute to vascular smooth muscle dysfunction and elevated peripheral resistance. In York platelet syndrome, a gain-of-function mutation in STIM1 underlies thrombocytopenia and abnormal platelet reactivity [94]. In type 2 diabetes, enhanced STIM1 activity correlates with a prothrombotic state and increased cardiovascular risk [96]. In addition, recent studies suggest that empagliflozin inhibits InsP3-induced ER Ca^2+^ release and SOCE in atrial fibroblasts, supporting the idea that modulation of intracellular Ca^2+^ handling may be beneficial in limiting cardiac fibrogenesis [105]. Collectively, these findings identify STIM1 and Orai1 as important molecular nodes in cardiovascular pathophysiology and potential therapeutic targets for limiting cardiovascular fibrosis.

### 4.2. Kidney Fibrosis

In the kidney, dysregulation of STIM1/Orai1-mediated SOCE has been linked to renal fibrosis, a common pathological endpoint of chronic kidney disease [106]. Recent studies have shown that Orai1 promotes Th17 cell activation and contributes to renal injury [90]. In mice fed a high-fat diet, Orai1 also enhances extracellular matrix synthesis in proximal tubular cells and promotes renal fibrosis [87]. Moreover, kidney biopsies from patients with fibrotic nephropathies show increased Orai1 expression in proximal tubular epithelial cells [107]. Increased STIM1 and Orai1 expression may activate injured tubular epithelial cells and interstitial fibroblasts, leading to sustained Ca^2+^ influx and increased production of pro-fibrotic mediators such as transforming growth factor-β1 (TGF-β1). These events in turn promote fibroblast-to-myofibroblast differentiation, excessive extracellular matrix deposition, and progressive disruption of normal kidney architecture.

### 4.3. Pulmonary Fibrosis

In the lung, aberrant STIM1/Orai1-mediated SOCE has been implicated in both idiopathic pulmonary fibrosis (IPF) and fibrosis secondary to chronic lung injury. Enhanced SOCE activity can increase the production of the pro-fibrotic cytokine TGF-β1 and activate downstream effectors including NFAT and Smad signaling. Recent studies have shown that SOCE-mediated Ca^2+^ signaling promotes lung fibroblast activation, including proliferation, contraction, migration, and collagen production [22]. In vivo and in vitro studies further indicate that Orai3 knockdown reduces multiple TGF-β1-induced fibroblast responses, including proliferation, α-SMA expression, extracellular matrix production, and activation of NFAT1 and Calpain/ERK signaling pathways [85]. Moreover, beractant has been reported to increase intracellular Ca^2+^ through ER Ca^2+^ release and SOCE, thereby promoting anti-fibrogenic responses and apoptosis in normal human lung fibroblasts [108].

### 4.4. Liver Fibrosis

Proper control of cytosolic Ca^2+^ in hepatic stellate cells is essential for hepatic fibrogenesis [109]. In non-alcoholic fatty liver disease, Ca^2+^ imbalance is considered one of the factors contributing to hepatic steatosis [110]. Lipid accumulation in hepatocytes has been reported to alter Ca^2+^ signaling, thereby promoting steatosis and progression to non-alcoholic steatohepatitis (NASH) [111]. In addition, several studies suggest that lipid accumulation suppresses SOCE, which may further aggravate intracellular lipid accumulation [89,112]. These findings raise the possibility that restoration of SOCE or normalization of Ca^2+^ homeostasis may help limit lipid accumulation, hepatic stellate cell activation, and fibrotic progression in chronic liver disease.

### 4.5. Other Systemic Diseases

Although fibrosis is most evident in classic organ systems such as the heart, kidney, lung, and liver, dysregulated SOCE also contributes to other disease states in which chronic inflammation, stromal activation, or pathological tissue remodeling are prominent. These broader contexts further support the concept that STIM/Orai signaling controls core cellular programs relevant to fibrotic progression.

#### 4.5.1. Cancers

A large body of evidence indicates that increased SOCE and elevated STIM1/Orai1 expression contribute to the progression of multiple cancers, including hepatic, pulmonary, ovarian, mammary, colorectal, and gastric carcinomas, as well as multiple myeloma [101,113]. Clinically, elevated Orai1 expression in oesophageal squamous cell carcinoma is associated with worse overall survival and recurrence-free survival [102]. STIM1 has also been shown to promote cervical cancer progression by driving vascular endothelial growth factor (VEGF) production, thereby facilitating tumor angiogenesis and growth. Moreover, increased STIM1 expression in primary cervical tumors correlates with aggressive clinical features, including larger tumor size and increased lymph node metastasis, suggesting poor prognosis [103]. Importantly, SOCE also contributes to desmoplastic stromal responses and fibroblast activation within the tumor microenvironment, thereby linking cancer-associated SOCE signaling to fibrosis-related remodeling.

#### 4.5.2. Immunodeficiency Disorders

Ca^2+^ influx mediated by CRAC channels serves as a critical biochemical link between antigen recognition and lymphocyte effector function. Following antigen stimulation, sustained Ca^2+^ entry activates NFAT-dependent transcriptional programs that are required for cytokine production, clonal expansion, differentiation, and immune effector responses. In patients lacking functional Orai expression, immunodeficiency is dominated by defective T-cell activation, and possibly impaired B- and NK-cell activation, whereas lymphocyte development remains largely intact [99,114]. Although this disease category is not fibrotic per se, it underscores the central importance of STIM/Orai signaling in controlling cell fate and downstream transcriptional responses that are also relevant to chronic inflammatory and fibrotic disorders.

#### 4.5.3. Neurodegenerative Diseases

Ca^2+^ acts as an important charge carrier that supports neuronal rhythmicity under certain conditions and influences both basic neuronal physiology and higher-order circuit functions, including sensory processing and memory formation. Orai and STIM proteins have been implicated not only in normal brain function but also in neurological disorders such as Alzheimer’s disease and epilepsy [11]. While the connection to fibrosis is less direct, these observations further highlight the broad pathological relevance of dysregulated SOCE.

Collectively, the findings summarized above indicate that aberrant STIM/Orai-mediated SOCE regulates a common set of pathological processes, including chronic inflammation, uncontrolled proliferation, and pathological tissue remodeling. Notably, SOCE appears to participate in fibrogenesis across multiple organs, regardless of the initiating insult. In the cardiovascular system, it contributes to vascular dysfunction and fibrotic remodeling. In the kidney, it promotes tubular injury and interstitial matrix deposition. In the lung, it supports myofibroblast differentiation and progressive destruction of alveolar structure. In the liver, it influences hepatic stellate cell activation and chronic fibrotic remodeling. Together, these findings suggest that STIM1/Orai1-mediated Ca^2+^ signaling functions as a common molecular hub that integrates pro-fibrotic stimuli and coordinates a conserved fibrogenic program. The following sections, therefore, discuss in greater detail the downstream signaling mechanisms, regulatory factors, and therapeutic strategies targeting SOCE in fibrosis.

## 5. SOCE in Fibrosis: Mechanisms and Therapeutic Opportunities

Fibrosis is a major contributor to global morbidity and mortality, and it serves as a common pathological endpoint for a variety of chronic inflammatory diseases. It is due to a dysregulated tissue repair process, which involves excessive extracellular matrix deposition, caused by abnormal responses to injury and connective tissue damage [115]. Fibroblasts are central to this response because they normally maintain and repair connective tissue. Their differentiation into hyperproliferative, matrix-secreting, and contractile myofibroblasts is a critical event in fibrotic progression [116,117]. In addition, myofibroblast heterogeneity and the acquired resistance of these cells to apoptosis during disease progression present major therapeutic challenges [118]. By progressively disrupting normal tissue architecture, fibrosis frequently advances to organ failure and contributes substantially to disability and death worldwide.

### 5.1. Current Fibrosis Therapeutics

Transforming growth factor-β (TGF-β) is a key pro-fibrotic cytokine and a central mediator of fibrosis, and it is, therefore, considered a promising therapeutic target in fibrosis-related diseases. Current strategies mainly aim to suppress TGF-β production or activity, including nucleic acid-based approaches designed to inhibit TGF-β synthesis [119].

Pirfenidone (PFD) is an orally administered synthetic small molecule that inhibits TGF-β at both the transcriptional level and the level of biological activity [120,121]. It also suppresses fibroblast proliferation and reduces the synthesis of type I and type III collagen [122]. GSK3008348 is a small-molecule inhibitor that specifically targets integrin αvβ6. By engaging this integrin, it inhibits TGF-β activation with sustained duration and reduces both pulmonary collagen deposition and serum C3M levels [123]. SM04646 is an inhaled small-molecule inhibitor of the WNT pathway that has been developed for the treatment of idiopathic pulmonary fibrosis (IPF). It suppresses TGF-β-induced extracellular matrix gene expression and inhibits myofibroblast differentiation [124]. Omipalisib (GSK2126458), a potent and selective inhibitor of PI3Kα and mTOR signaling, was initially developed for cancer therapy and was later found to attenuate fibroblast proliferation and TGF-β-induced collagen synthesis in primary human lung fibroblasts [125].

However, currently available anti-fibrotic therapies often require relatively high drug doses and may be associated with systemic side effects [126,127]. Long-term use of pirfenidone, for example, has been linked to adverse effects including gastrointestinal and neurological complications [128]. In many cases, these adverse effects arise because upstream signaling molecules participate in multiple biological pathways, and their nonspecific inhibition may, therefore, lead to systemic toxicity [129]. These limitations highlight the urgent need for new anti-fibrotic therapies that are both more selective and less toxic.

Given that fibrosis is driven by persistent activation of Ca^2+^-dependent pathways controlling fibroblast differentiation, inflammatory signaling, and extracellular matrix production, SOCE has emerged as an attractive mechanistic target. Unlike broader upstream anti-fibrotic strategies, targeting STIM1/Orai1-mediated Ca^2+^ entry may allow more selective interruption of the intracellular signaling programs that sustain myofibroblast activation and pathological tissue remodeling.

### 5.2. STIM1/Orai1-Mediated Ca^2+^ Signaling Pathway in Fibrosis

**Figure 3:**
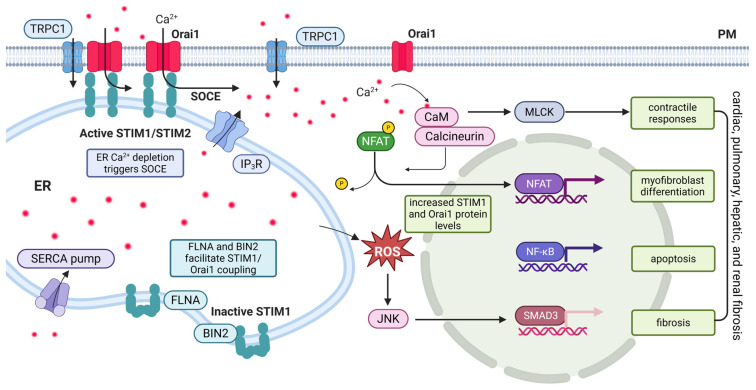
STIM1/Orai1-mediated Ca^2+^ signaling pathway in fibrosis. Upon endoplasmic reticulum (ER) Ca^2+^ store depletion, STIM1 is activated and couples to Orai1 channels at ER–plasma membrane (PM) junctions to trigger store-operated Ca^2+^ entry (SOCE). The resulting increase in cytoplasmic Ca^2+^ activates multiple downstream pro-fibrotic signaling pathways, including the CaM/MLCK axis, CaN/NFAT signaling, NF-κB activation, and the ROS–JNK/p38–SMAD3 pathway, thereby promoting myofibroblast activation and fibrotic remodeling. Abbreviations: ER, endoplasmic reticulum; TRPC1, transient receptor potential canonical 1; IP_3_R, inositol 1,4,5-trisphosphate receptor; SERCA, sarcoplasmic/endoplasmic reticulum Ca^2+^-ATPase; SOCE, store-operated Ca^2+^ entry; STIM1, stromal interaction molecule 1; PM, plasma membrane; CaM, calmodulin; MLCK, myosin light-chain kinase; NFAT, nuclear factor of activated T cells; NF-κB, nuclear factor kappa-B; SMAD3, SMAD family member 3; JNK, c-Jun N-terminal kinase; p38, p38 mitogen-activated protein kinase. Figure created with BioRender.com.

Current therapeutic options for organ fibrosis remain limited, underscoring the importance of identifying new molecular targets. In this context, STIM1/Orai1-mediated Ca^2+^ signaling has emerged as a central pathway linking extracellular stimuli to fibrogenic cellular responses. As outlined in Figure 3, this pathway can be divided into two major modules: upstream ER Ca^2+^ depletion and SOCE activation, and downstream cytoplasmic Ca^2+^-dependent signaling cascades.

Upon depletion of ER Ca^2+^ stores, STIM1 and STIM2 become activated. Activated STIM proteins interact with Orai1 to trigger classical store-operated Ca^2+^ entry, thereby enabling Ca^2+^ influx from the extracellular space into the cytoplasm. In addition to this canonical STIM/Orai pathway, some studies indicate that STIM1, Orai1, and TRPC1 can form a distinct SOC channel complex in certain cellular contexts, in which Orai1-mediated local Ca^2+^ entry may facilitate TRPC1 recruitment and/or activation [130,131]. Additional factors, including IP_3_R-mediated ER Ca^2+^ release and BIN2- or FLNA-enhanced STIM1/Orai1 coupling, can further promote SOCE [132,133].

Among the downstream pathways activated by elevated cytoplasmic Ca^2+^, several appear to be particularly relevant to fibrotic signaling. First, in the CaM/CaN–NFAT axis, Ca^2+^ binds to calmodulin (CaM) [134] and enhances myosin light-chain kinase (MLCK) expression, thereby facilitating smooth muscle contraction [135,136]. At the same time, Ca^2+^-bound CaM activates calcineurin (CaN), which dephosphorylates NFAT and promotes its translocation into the nucleus, where it drives pro-fibrotic transcriptional programs and myofibroblast differentiation [137]. Second, in the NF-κB pathway, elevated STIM1/Orai1 expression can enhance NF-κB signaling, thereby promoting inflammation and apoptosis and contributing to myocardial hypertrophy and fibrosis [138]. Third, in the ROS–JNK–SMAD3 axis, elevated cytoplasmic Ca^2+^ promotes activation of ROS-generating enzymes and accumulation of reactive oxygen species (ROS) [139,140]. ROS accumulation in turn activates the JNK/p38 MAPK signaling cascade [141,142], which modulates R-SMAD phosphorylation and influences SMAD nuclear translocation and DNA binding [143,144]. Because SMAD3 is a direct downstream effector of TGF-β1, this signaling axis provides an important mechanistic link between SOCE and fibrotic gene expression [145,146].

Together, these findings support the concept that STIM1/Orai1-mediated Ca^2+^ influx is not merely a passive consequence of ER store depletion but rather a central signaling hub that amplifies multiple pro-fibrotic pathways.

### 5.3. Pharmacological Modulators of SOCE

**Figure 4:**
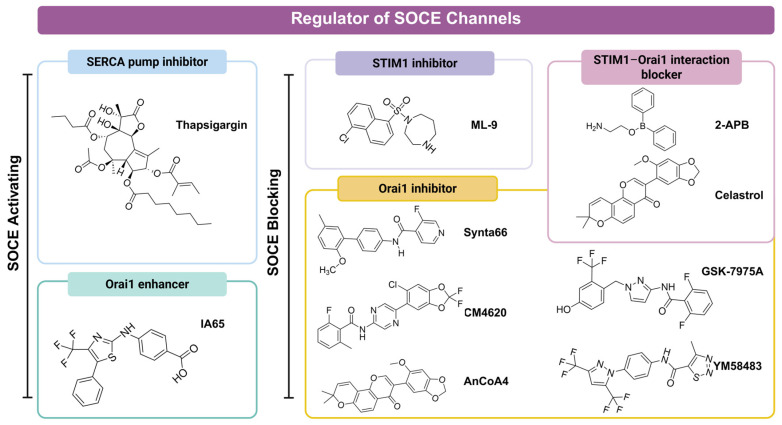
Small molecule regulators of the SOCE pathway, categorized into two groups: SOCE activating and SOCE blocking agents.

A variety of small molecules have been reported to modulate SOCE by targeting STIM1, Orai proteins, or the interaction between them. Based on their functional effects, these SOCE modulators can be broadly classified into two categories: SOCE activators and SOCE blockers (Figure 4).

#### 5.3.1. SOCE Activating Agents

Thapsigargin (TG) is a naturally occurring compound originally isolated from the roots and fruits of Mediterranean plants of the *Thapsia* genus [147]. It functions as a potent and specific inhibitor of the SERCA pump. By blocking SERCA activity, TG depletes ER Ca^2+^ stores, thereby increasing cytoplasmic Ca^2+^ levels and indirectly activating SOCE. Multiple studies have established that inhibition of SERCA is the primary pharmacological action of TG [148]. Because its effect is mediated through ER store depletion, TG is mainly used as an experimental tool to activate SOCE rather than as a therapeutic strategy.

IA65 is an Orai1 enhancer that directly increases Orai1 channel activity and promotes Ca^2+^ influx, with relatively high selectivity for Orai1 and minimal effects on Orai2 and Orai3. Recent studies have shown that IA65 potentiates Orai1 function not only in heterologous expression systems but also in vascular smooth muscle cells and skeletal muscle fibers [149].

#### 5.3.2. SOCE Blocking Agents

In this review, SOCE blockers are categorized into three main groups: STIM inhibitors, Orai inhibitors, and STIM1–Orai interaction blockers. Among known pharmacological agents, ML-9 is one of the few compounds reported to target STIM1 rather than Orai1, supporting the concept that STIM1 activation can be selectively modulated to regulate CRAC channel activity [150].

Several Orai-directed inhibitors have also been identified. These include Synta66, a relatively selective CRAC channel inhibitor with differential effects on Orai isoforms [151]; CM4620, which is a small-molecule CRAC/Orai channel inhibitor that preferentially inhibits Orai1/STIM1-mediated Ca^2+^ entry, with reported lower potency toward Orai2/STIM1-mediated currents, and has been shown to reduce inflammation and disease severity in preclinical models of acute pancreatitis [152,153]; AnCoA4, which blocks CRAC channels and attenuates T-cell activation both in vitro and in vivo by reducing Orai1 recruitment into puncta and inhibiting constitutively active Orai1 V102C channels [154]; GSK-7975A, which inhibits Orai1 and Orai2 without affecting STIM1 oligomerization or STIM1–Orai1 interaction [155]; and YM-58483, which is a potent Orai1 inhibitor that has demonstrated analgesic effects in neuropathic pain models [156]. In addition, classic SOCE modulators, such as 2-APB and celastrol, can disrupt the interaction between STIM1 and Orai1, thereby inhibiting SOCE [157,158,159].

Notably, several SOCE blockers have also shown promise in experimental models relevant to fibrosis. For example, treatment of systemic sclerosis fibroblasts with the SOCE inhibitor 2-APB selectively reduces fibrosis markers and alters cell morphology [160]. Similarly, although TGF-β1 and vitamin C promote collagen deposition in pancreatic stellate cells, the Orai1 inhibitor Synta-66 suppresses Ca^2+^ influx and reduces collagen release from primary murine pancreatic stellate cells [84]. In addition, Ang II-induced upregulation of fibronectin (FN), connective tissue growth factor (CTGF), and smooth muscle α-actin (α-SMA) is attenuated by the SOCE inhibitor SKF-96365. Similar anti-fibrotic effects are also observed following knockdown of STIM1 and Orai1 [81].

## 6. Conclusions and Perspective

Accumulating evidence indicates that pharmacological inhibition of store-operated calcium entry (SOCE) holds considerable promise as a therapeutic strategy for fibrotic diseases. A number of SOCE inhibitors, including 2-APB, Synta-66, and SKF-96365, have been shown to attenuate key fibrotic processes in a variety of experimental models. By targeting dysregulated Ca^2+^ signaling, these compounds can suppress fibrosis-associated markers such as α-SMA and connective tissue growth factor (CTGF), reduce pathological collagen deposition, and inhibit myofibroblast activation.

However, translating SOCE-targeted therapies into clinical practice will require careful consideration of several important challenges. First, many currently available SOCE modulators still lack sufficient selectivity and may affect multiple Ca^2+^-handling pathways, thereby limiting therapeutic specificity. The development of compounds with greater selectivity for disease-relevant SOCE components, particularly Orai1 and STIM1 isoforms or variants enriched in fibrotic tissues, may improve efficacy while minimizing off-target effects. Second, because Ca^2+^ signaling participates in both physiological tissue repair and pathological remodeling, defining the optimal therapeutic window will be essential. The efficacy of intervention may differ substantially between the early phase of fibrotic activation and late-stage established fibrosis. Third, the contribution of distinct STIM and Orai isoforms may vary across organs, cell types, and stages of disease, suggesting that a more refined and context-dependent targeting strategy will likely be required. Finally, SOCE inhibitors may prove most effective when used in combination with existing anti-fibrotic therapies, such as inhibitors of the TGF-β pathway, thereby providing complementary or potentially synergistic suppression of fibrogenic signaling.

An important direction for future investigation is to define whether SOCE functions merely as a permissive Ca^2+^ entry pathway in fibrosis or instead acts as a central signaling hub that actively determines fibroblast state transitions. It is tempting to speculate that distinct STIM/Orai complexes may operate in a cell type-specific manner to control epithelial injury responses, fibroblast-to-myofibroblast differentiation, inflammatory activation, and extracellular matrix remodeling. One possibility is that different fibrotic organs rely on partially distinct SOCE signaling modules, such that the dominant pro-fibrotic pathway in the lung, kidney, heart, or liver is shaped by the local expression pattern of STIM and Orai isoforms, their accessory proteins, and the surrounding cytokine milieu. We further hypothesize that sustained SOCE may not only amplify canonical pro-fibrotic pathways, such as TGF-β/SMAD, NFAT, and NF-κB signaling, but may also help stabilize a persistent pathogenic cellular memory that promotes chronic myofibroblast activation and resistance to apoptosis. It will also be important to determine whether selective interruption of SOCE in specific stromal or epithelial cell populations can uncouple pathological fibrosis from physiological tissue repair.

In summary, targeting SOCE represents a mechanistically grounded strategy for disrupting core pro-fibrotic signaling pathways. Further progress in this area will require continued preclinical optimization, improved isoform and tissue selectivity, and rigorous early-phase clinical studies to evaluate safety, efficacy, and translational potential across diverse fibrotic disorders. Ultimately, successful clinical translation will likely depend on identifying the SOCE components most relevant to diseased tissues, achieving tissue-selective modulation, and defining the cellular and pathological contexts in which SOCE-targeted intervention provides the greatest anti-fibrotic benefit.

## Data Availability

No new data were generated or analyzed in this study. Data sharing is not applicable to this article.
